# A Systematic Review and Meta-Analysis of an Angiotensin Receptor–Neprilysin Inhibitor in Patients Using a Durable Left Ventricular Assist Device

**DOI:** 10.3390/jcm13247789

**Published:** 2024-12-20

**Authors:** Elfatih A. Hasabo, Burce Isik, Ammar Elgadi, Magdi S. Yacoub, Mohamed S. Bakr, Mohammed Mahmmoud Fadelallah Eljack, Sherif Sultan, Kadir Caliskan, Osama Soliman

**Affiliations:** 1 School of Medicine, College of Medicine Nursing and Health Sciences, University of Galway, H91 TK33 Galway, Ireland; elfatih.ahmed.hasabo@gmail.com (E.A.H.); magdisyacoub@gmail.com (M.S.Y.); 2 Discipline of Cardiology, Saolta Healthcare Group, Galway University Hospital, Health Service Executive and University of Galway, H91 YR71 Galway, Ireland; 3 Corrib-Curam-Vascular Group, University of Galway, H91 YR71 Galway, Ireland; 4 School of Medicine, University of Limerick, V94 T9PX Limerick, Ireland; 21149747@studentmail.ul.ie; 5 Faculty of Medicine, University of Khartoum, Khartoum 11115, Sudan; ammartarig56@gmail.com; 6 Faculty of Medicine, New Mansoura University, New Mansoura 35516, Egypt; 7 Faculty of Medicine, University of Bakht Alruda, Ad Duwaym 11112, Sudan; 8 Department of Vascular and Endovascular Surgery, Western Vascular Institute, University College Hospital, H91 YR71 Galway, Ireland; 9 Thoraxcenter, Department of Cardiology, Cardiovascular Institute, Erasmus MC University Medical Center, 3015 GD Rotterdam, The Netherlands; 10 Euro Heart Foundation, 2501 CH Den Haag, The Netherlands

**Keywords:** angiotensin receptor–neprilysin inhibitor, ventricular assist device, systematic review: meta-analysis, efficacy, safety

## Abstract

**Introduction:** Sacubitril/valsartan is an angiotensin receptor–neprilysin inhibitor (ARNI) drug used to treat patients with heart failure and has shown improvement in outcomes. Different studies reported the use of an ARNI in patients using left ventricular assist devices (LVADs). However, there are limited data on the use of ARNIs in this population. We aimed to assess the efficacy of ARNIs in LVAD patients. **Methods:** A systematic search was performed in PubMed, Scopus, Web of Science, Embase, and Cochrane from inception to November 2024. We used all relevant words for “ARNI” and “LVAD” to search, and we included studies that assessed ARNIs in patients with LVAD. Efficacy and safety outcomes were extracted from the included studies. R software version 4.4.2 was used for a meta-analysis. **Results:** Seven studies totaling 249 patients were included. The ARNI was found to be effective in improvements from baseline in the New York Heart Association (NYHA), B-type natriuretic peptide (BNP) (mean = −630.07 pg/mL, 95% CI [−1113.13, −147.01]), diuretic dose (furosemide equivalents) (mean= −76.05 mg/day, 95% CI [−145.11, −6.99]), left ventricular end-diastolic diameter (LVEED) (mean = −7.3 mm, 95% CI [−11.4, −3.1]), and left ventricular ejection fraction (LVEF) (mean =5, 95% CI [3.52, 6.48]). No improvement was found in the creatinine (Cr) level. However, a slight increase in the potassium level was noticed (mean= 0.17 mEq/L, 95% CI [0.02, 0.34]). The overall mortality in patients using the ARNI was 5%, 95% CI [0.00, 20], and discontinuation was found in 25%, 95% CI [0, 100]. **Conclusions:** The ARNI improved several cardiac structural and hemodynamic parameters in patients on LVAD support.

## 1. Introduction

The ARNI sacubitril–valsartan is being used for the treatment of heart failure with promising therapeutical effects across different cardiovascular patient populations. McMurray et al. demonstrated a 20% relative risk reduction in cardiovascular-related death and a 21% relative risk reduction in hospitalization secondary to heart failure when treated with sacubitril/valsartan vs. enalapril in patients with heart failure with a reduced ejection fraction (HFrEF) [1], highlighting its therapeutic superiority over angiotensin-converting enzyme inhibitors (ACE-Is) in the HFrEF population. Current standard medical therapy for LVAD patients includes the usage of anticoagulation and antiplatelet therapy, such as warfarin and aspirin, and heart failure medications. It may also include antiarrhythmics for managing arrhythmias [2].

The myocardial stretch, caused by the combination of a vasopressin, renin–angiotensin–aldosterone system (RAAS), and sympathetic nervous system-induced increase in preload and afterload, triggers the production of (BNP), which aids in natriuresis, decreased cardiac wall stress, and other reverse remodeling effects [3,4]. Sacubitril/valsartan has a dual mechanism of action. Sacubitril inhibits neprilysin, which breaks down natriuretic peptides and angiotensin II. This would lead to reduced natriuretic peptide degradation and bolster the effects of natriuresis. However, valsartan combats the effects of angiotensin II, which eventually helps patients using an LVAD [3,4].

Limited data exist on using ARNI in the LVAD population and whether it improves cardiac outcomes compared to standard-of-care medical treatments. This area requires further attention to close the knowledge gap and establish guidelines on using sacubitril/valsartan in the LVAD patient population. A retrospective study assessed the outcomes of patients post-LVAD implantation who received sacubitril/valsartan and showed a reduction of 8 mmHg in the mean arterial pressure (MAP) and reduced hospitalization due to heart failure [5]. The ENVAD-HF assessed sacubitril/valsartan in 60 LVAD patients, while SEAL-IT clinical trials compared the safety and efficacy of sacubitril/valsartan to the standard of care in 50 patients supported by LVAD; both trials are ongoing, and the results are not published yet [6,7].

Two case series studies by Randhawa et al., 2020 and Goldberg et al., 2021 have also demonstrated the safety and efficacy of sacubitril/valsartan in the LVAD patient population. Randhawa et al., 2020, included ten patients with continuous flow-LVAD and showed a significant reduction in the MAP, while Goldberg et al., 2021, included 21 participants using LVAD and showed an improvement in the diuretic dose requirement and LVEDD [8,9].

To date, clinical trials have not published the results of using ARNI in patients of LVAD. Furthermore, ARNI has shown its efficacy in patients with heart failure in the PARADIGM-HF trial [1]. Few studies reported data on using ARNI using an LVAD, which raises the need for addressing and filling this gap in knowledge; therefore, this systematic review and meta-analysis aimed to summarize the existing knowledge and synthesize the data on the cardiac recovery outcomes of patients on sacubitril/valsartan supported by an LVAD.

## 2. Methods

### 2.1. Methods and Materials

We conducted a systematic review and meta-analysis of the efficacy and safety of ARNIs among patients using an LVAD according to the Cochrane Handbook for systematic reviews of interventions [10]. This systematic review was reported using the preferred reporting items for systematic review and meta-analysis (PRISMA statement) [11]. The study was started before the protocol registration; therefore, this review was not eligible for PROSPERO protocol registration.

#### 2.1.1. Search Strategy

We searched PubMed, Scopus, Web of Science, Embase, and Cochrane databases by using the following keywords: (Entresto OR “sacubitril/valsartan” LCZ696 OR Azmarda OR Neparvis OR angiotensin receptor/neprilysin inhibitor OR ARNI) AND (“left ventricular assist device” OR LVAD OR heartmate OR heartware OR “heart pump” OR Jarvik-2000 OR Thoratec OR “ventricular assist device” OR vad OR “Mechanical Circulatory Support” OR mcs OR “biventricular assist device” OR bivad OR Heart Assist Device OR novacor OR “left ventricular assist system” OR LVAS), from inception until November 2024 without any limitation in time. Details of studies yielded from searches in databases are found in Supplementary Table S1.

**Eligibility Criteria and Study Selection:**

In this systematic review, we included all studies with the following criteria:

1. Randomized control trials (RCTs), case reports, cohort studies, case-control studies, or observational studies that included patients using ARNIs in patients using an LVAD.
2. Studies in the English language.

This study excluded review articles, opinion papers, systematic reviews, case reports, conference abstracts, and study protocols or any study not in the English language.

The reviewers independently screened all the studies retrieved from databases during the title and abstract screening. The remaining studies from the title and abstract screening were included in the full-text screening. Then, full-text screening was performed independently. During the title and abstract or full-text screening, any discrepancy between reviewers was discussed and resolved by a third reviewer before the final inclusion of the study.

#### 2.1.2. Data Extraction

The authors extracted the following data from the included studies:

1. Baseline information and summary of included studies.
2. Efficacy of ARNI in patients using LVAD: several outcomes were extracted from included studies, such as the change from baseline to follow-up in LVEDD, LVEF, BNP, MAP) diuretic dose (furosemide equivalents), serum Cr, serum potassium, and other outcomes extracted from the studies and not included in the meta-analysis.
3. Safety and outcomes of ARNI in patients using LVAD: mortality and discontinuation.

#### 2.1.3. Quality Assessment

We assessed the quality using the Newcastle–Ottawa Scale (NOS) for cohort studies and the adapted NOS [12]. The NOS consists of the following questions: “representativeness of the exposed cohort”, “selection of the non-exposed cohort”, “ascertainment of exposure”, and “demonstration that outcome of interest was not present at start of study” (selection category consists of three questions), “comparability of cohorts on the basis of the design or analysis” (comparability category consists of one question) and “assessment of outcome”, “was follow-up long enough for outcomes to occur”, and “adequacy of follow up of cohorts” (outcomes category consists of three questions). For the single-arm studies, the total score for the adapted NOS is six after excluding the “selection of the non-exposed cohort” and “Comparability of cohorts based on the design or analysis”. For studies with a control group, scores of 7–9, 4–6, and <4 were classified as having a low, moderate, or high risk of bias, respectively. Single-arm studies with scores more than 4 are considered a low risk of bias.

#### 2.1.4. Data Analysis

Data were extracted from the included studies and presented as tables and figures. Efficacy outcomes were presented as the mean and 95% confidence interval (CI), and safety outcomes were presented as the proportion and 95% CI to be presented as forest plots. The I-square (I^2^) test was used to assess the heterogeneity, and the random effect model was used in the presence of heterogeneity. R software version 4.4.2 was used for the meta-analysis.

## 3. Results

### 3.1. Literature Search

**Figure 1:**
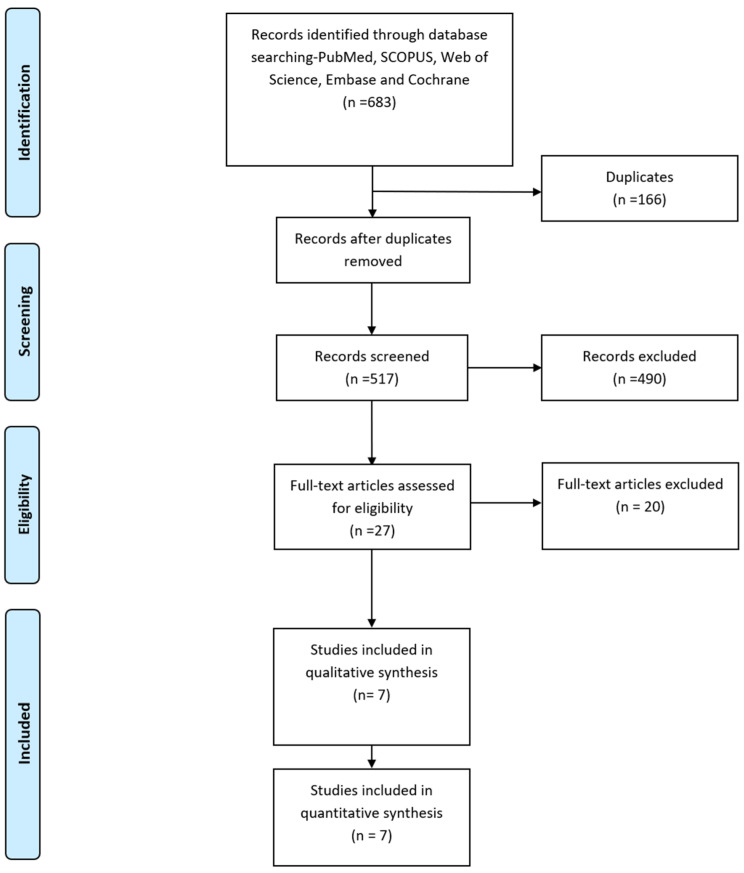
PRISMA Flow diagram.

The PRISMA Flow diagram in Figure 1 describes the literature search conducted in this study. We identified six hundred and seventy-nine relevant publications after searching PubMed, SCOPUS, Web of Science, Embase, and Cochrane. After removing duplicates, 538 publications remained. Seven studies that met our inclusion criteria [8,9,13,14,15,16,17] were included in the qualitative and quantitative analysis.

### 3.2. Summary and Baseline Data of Included Studies

Two hundred and forty-nine patients from seven complete text studies [8,9,13,14,15,16,17] were included in our meta-analysis. Entresto was the ARNI used in the included studies. Table 1 summarizes the baseline clinical data of the included studies, and Table 2 lists the risk factors and types of devices used among participants. Table 3 lists the summary of all included studies. Table 4 lists the summary of the effect of an ARNI in patients with an LVAD.

### 3.3. Quality Assessment of Included Studies

The quality assessment using the adapted NOS and Cochrane Risk of Bias tool showed a low risk of bias among the included studies. Supplementary Table S2 provides further details about the assessment of quality for the included studies.

### 3.4. Efficacy of ARNI

1. Change from baseline in LVEDD (mm)

**Figure 2:**
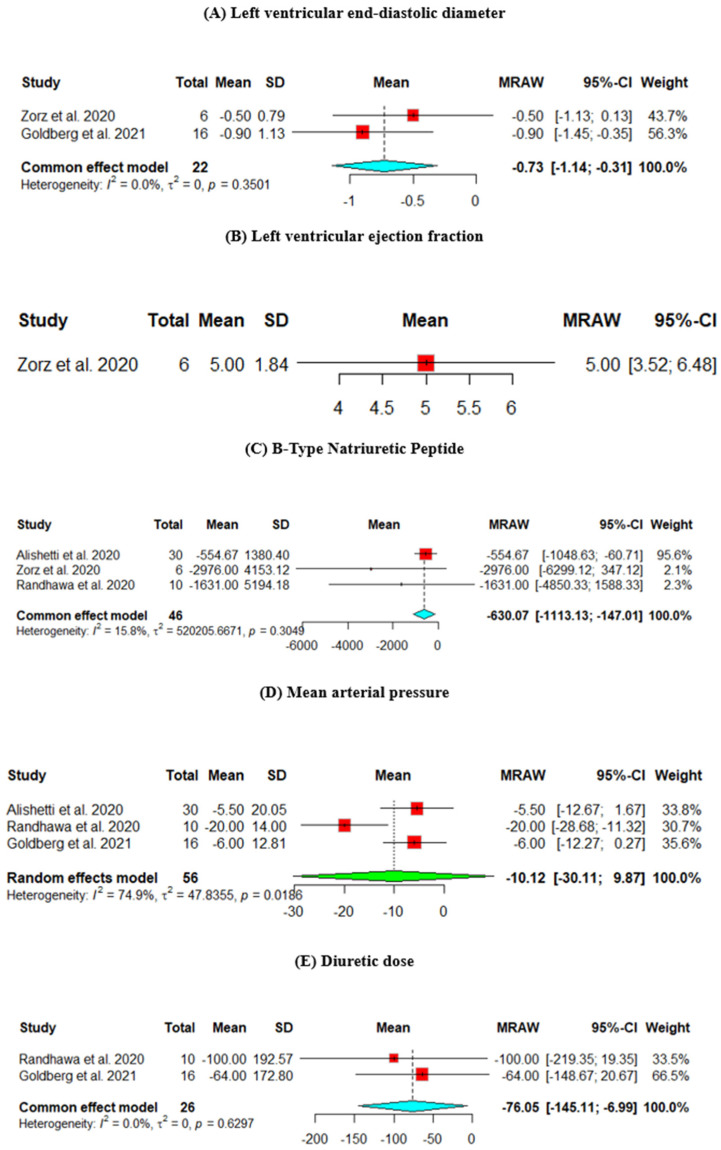

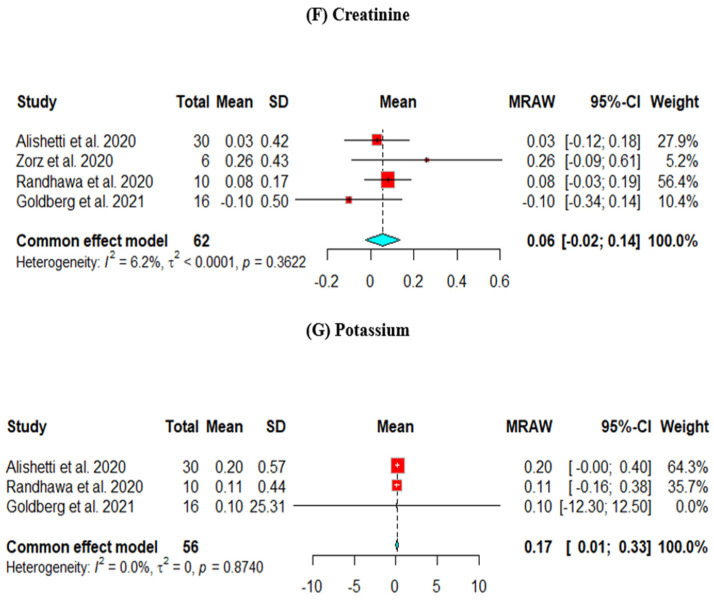
Efficacy outcomes of the change from baseline in the (**A**) left ventricular end-diastolic diameter, (**B**) left ventricular ejection fraction, (**C**) B-type natriuretic peptide, (**D**) mean arterial pressure, (**E**) diuretic dose, (**F**) creatinine, and (**G**) potassium (Goldberg et al., 2021 [8], Randhawa et al., 2020 [9], Alishetti et al., 2020 [13], Zorz et al., 2020 [17]).

The change from baseline LVEDD was assessed across two studies. The heterogeneity across the studies is *p* = 0.3501, I^2^ = 0%. The results showed a significant decrease in the LVEDD (mean = −7.3, 95% CI [−11.4, −3.1]) (Figure 2A).

2. Change from baseline in LVEF (%)

The change from baseline LVEF was assessed in one study, which showed an improvement in the EF (mean = 5, 95% CI [3.52, 6.48]) (Figure 2B).

3. Change from baseline in BNP (pg/mL)

The change from baseline in the BNP was assessed across three studies. The results showed a statistically significant decrease in the BNP level (mean = −630.07, 95% CI [−1113.13, −147.01]) (Figure 2C).

4. Change from baseline in MAP (mmHg)

The change from baseline MAP was assessed across three studies. The heterogeneity across the studies is *p* = 0.0186, I^2^ = 74.9%, and the results did not show a significant decrease in the MAP (mean = −10.12, 95% CI [−30.11, 9.87]) (Figure 2D).

5. Change from baseline in Diuretic dose (furosemide equivalents) (mg)

The change from baseline in the diuretic dose was assessed across two studies. The results showed a statistically significant decrease in the diuretic dose (mean = −76.05, 95% CI [−145.11, −6.99]) (Figure 2E).

6. Change from baseline in Cr (mg/dL)

The change in Cr from baseline was assessed across four studies. The results did not show a significant decrease in the Cr level (mean = −0.06, 95% CI [−0.02, 0.14]) (Figure 2F).

7. Change from baseline in Potassium (mEq/L)

The change in potassium from baseline was assessed across four studies. The results showed a statistically significant increase in the potassium level (mean = 0.17, 95% CI [0.01, 0.33]) (Figure 2G).

### 3.5. Safety of ARNI

1. Mortality

**Figure 3:**
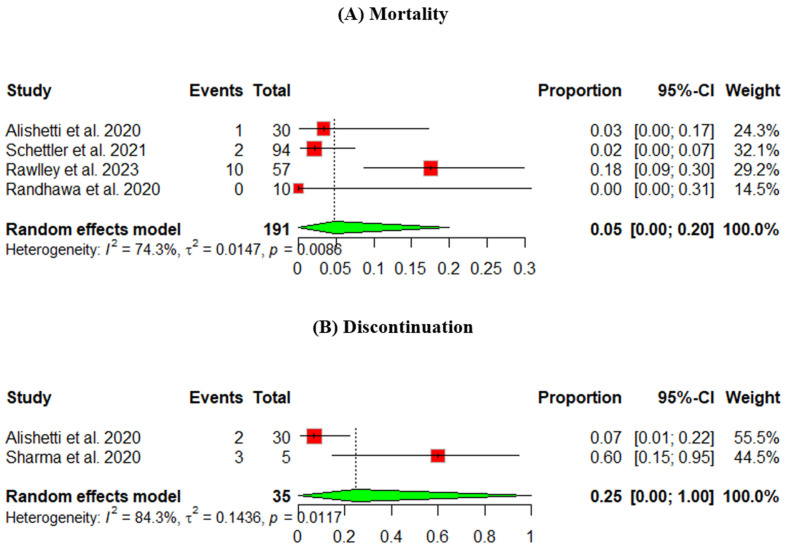
Safety outcomes of an ARNI in participants using an LVAD: (**A**) mortality, (**B**) discontinuation (Randhawa et al., 2020 [9], Alishetti et al., 2020 [13], Rawley et al., 2023 [14], Schnettler et al., 2021 [15], Sharma et al., 2020 [16]).

The mortality rate was measured across four studies. The heterogeneity across the studies is *p* = 0.0086, I^2^ = 74.3%. The pooled percentage of the mortality rate was 5%, 95% CI [0.00, 20] (Figure 3A).

2. **Discontinuation**

The discontinuation rate was measured across two studies, and the pooled percentage was 25%, 95% CI [0, 100] (Figure 3B).

## 4. Discussion

### 4.1. Main Findings

This study assessed an ARNI’s safety and effectiveness in patients using LVADs. Since most research on ARNIs has focused on heart failure in general, this study offers a thorough analysis of a more specialized population with LVADs. Thus, the findings of this study fill a significant gap in the literature.

Our findings demonstrate a notable improvement in cardiac function, fluid regulation, and hemodynamics concerning the effectiveness of ARNIs, which is reflected by an improvement in the LVEDD, BNP, MAP, and daily diuretic dose. All of these lead to improvements in heart failure symptoms and diuretic requirements in patients with an LVAD, which need further confirmation and studies in clinical trials.

The ARNI showed a significant decrease in LVEDD in this study. According to Castrichini et al. (2020) [18], this is explained by the induction of reverse remodeling, which improves left ventricular strain. However, the fact that Nowalk et al. (2019) [19] only included African American patients and had a smaller sample size may help to explain the results’ significant heterogenicity. Additionally, compared to the other three studies in our analysis, this study shows a more substantial decrease in the LVEDD. Nonetheless, a combined pharmacological analysis shows that the impact of an ARNI in patients with heart failure is not influenced by ethnicity [20]. Moreover, a study by Dandel et al., 2008 [21], confirmed that an LVEDD > 55 mm was found to be related to the reappearance of heart failure after LVAD explantation and that an ARNI will help to reduce this recurrence of heart failure.

Regarding BNP, the pooled analysis showed that the ARNI decreases BNP levels, likely due to decreased hemodynamic stress and neurohormonal activation [13]. BNP is a crucial biomarker in heart failure management due to its strong correlation with left ventricular unloading and the gradual improvement in clinical outcomes for end-stage heart failure patients fitted with an LVAD. Even minor decreases in BNP can indicate reduced ventricular strain and improved hemodynamics. However, the extent to which these changes affect long-term clinical outcomes, such as survival rates and quality of life, remains a key consideration.

For patients with existing renal damage, the minimal risk of kidney failure associated with ARNIs provides a significant advantage, offering a safer alternative compared to other medications [22,23]. Hence, while the observed changes in BNP are modest, they still hold potential clinical importance, especially in managing advanced heart failure in patients with an LVAD. A previous meta-analysis of ARNIs in heart failure patients and end-stage kidney disease revealed a positive effect of ARNI in improving the left ventricular function and no significant change in hyperkalemia [24], which could be also seen in patients with an LVAD.

Regarding controlling blood pressure in heart failure patients, MAP is the focus and assessed across the studies. Given that in our LVAD population, ARNI treatment decreased MAP but not significantly and it became in the normal range, ARNI use will be a suitable substitute for calcium channel blockers, as they may be contraindicated for individuals with a reduced LVEF [8,25]. Controlling MAP will help in adjusting pump flow and decrease the incidence of neurological complications [26].

The decrease in daily diuretic doses in our study reflects an additional feature. Another study showed that this decrease was independent of the improvement in blood pressure [8]. This will help these patients with heart failure and using an LVAD by improving their symptoms.

Pooled results did not show a significant change in serum creatinine. In contrast to serum potassium, there was a small but statistically significant increase in its level, which is numerically small. Alishetti et al. obtained a similar result [13]. However, a meta-analysis on the renal safety of ARNIs suggests a possible risk of increased serum creatinine and potassium; however, this risk is lower compared to ACE-i or an angiotensin II receptor blocker (ARB) [22].

Regarding the LVEF, only one study reported this outcome and showed significant improvement. Further studies are required in patients with LVADs to confirm this finding, and the results are confirmed by another study on heart failure with a reduced ejection fraction, which tended to benefit from ARNIs with a statistically significant increase in the ejection fraction [23]. Improvements in LVEF will eventually lead to meeting the criteria of LVAD explants, which was identified previously with an LVEF > 45% [27].

ARNI’s safety has been studied in most of its indications, and it was found to be safe and had fewer adverse events in heart failure patients [8]. In our analysis, some adverse events were reported. Most important is the pooled mortality, which is nearly 6%, which reflects the safety of ARNIs among this population. Rawlley et al., 2023, which included all-cause mortality, found the highest mortality, which was 17.5% and less than that in the ACE-i group [14].

This study offers compelling insights into the transformative potential of ARNIs in clinical practice, particularly for patients with LVAD. ARNIs present a crucial opportunity for patients with elevated BNP levels to enhance hemodynamic stability, effectively addressing their unique health needs. By incorporating ARNIs into treatment plans, we can significantly reduce reliance on diuretics, alleviate the medication burden, and ultimately improve patients’ quality of life.

Moreover, ARNIs are a valuable alternative to traditional calcium channel blockers for blood pressure management, providing healthcare providers with a more versatile toolkit for optimizing patient outcomes. The minimal impact on serum creatinine levels further distinguishes ARNIs from other treatments, such as ACE-is and ARBs, making it an attractive choice for patients concerned about renal health.

Implementing randomized clinical trials is imperative to reduce confounding variables and deliver robust evidence on the safety and efficacy of ARNIs in LVAD patients. This approach will ensure that we can confidently advocate for ARNIs as an effective treatment option in this population.

### 4.2. Strengths

Our meta-analysis is considered the first one investigating ARNIs in patients with LVADs. It provides valuable information and an in-depth understanding of the efficacy and safety of ARNIs in LVAD patients across several parameters. It also sets the stage for more targeted and robust future studies.

Further studies must incorporate multiple centers to enhance the diversity and representativeness of various populations. A larger sample size is essential to bolster the statistical power of the findings. Establishing a standardized follow-up period is crucial for evaluating long-term outcomes, such as survival and quality of life. Additionally, the detailed reporting of missing data and consistent outcome measurements across studies will minimize heterogeneity and facilitate better comparisons and meta-analyses.

### 4.3. Limitations

Some limitations are found in this systematic review. The published data about ARNIs in LVADs are scarce regarding publications and patient samples. Multicenter trials involving more patients are needed to investigate the effect of ARNIs on this population. Also, the included studies are limited by heterogeneity in the duration of patients’ follow-up and selected outcome variables in some studies. However, this was resolved by using the random effect model during the meta-analysis.

## 5. Conclusions

In conclusion, the ARNI showed a good efficacy and safety profile in patients with LVADs by improving hemodynamics, diuretic requirements, and BNP levels. Future randomized clinical trials need to be established to investigate and understand the effect of ARNIs in LVADs and investigate the long-term effect of this drug.

## Figures and Tables

**Table 1:** Baseline data of patients with LVAD using an ARNI.

Study	No. of Patients	Age (Years)	Male (%)	LVEF, Mean ± SD	Duration of LVAD Support	LVEDD, mm	MAP (mmHg)	NYHA Class
Alishetti et al., 2020 [13]	30	55.6 (43.3–69.8)	27 (90%)	-	-	-	91.5 (80–97)	-
Sharma et al., 2020 [16]	5	67 ± 6.9	4 (80%)	-	-	-	94 (86–100)	-
Zorz et al., 2020 [17]	6	58.3 ± 7.4	6 (100%)	33.5 ± 1.2	-	55 ± 7	-	-
Randhawa et al., 2020 [9]	10	58 ± 9	7 (70%)	-	-	-	98.8 ± 12.2	-
Schnettler et al., 2021 [15]	94	-	-	-	-	-	-	-
Goldberg et al., 2021 [8]	21	-	17 (81%)	-	19.6 ± 23 months	66 ± 8	89 ± 8	I: 1 (5%) II: 9 (43%) III: 7 (33%) IV: 4 (19%)
Rawley et al., 2023 [14]	83	53.1 ± 15.5	61 (73.5%)	-	-	-	-	-

Data are presented as the mean (SD), median (IQR), and number (percentage). Abbreviations: IQR, interquartile range; LVAD, left ventricular assist device; LVEDD, left ventricular end-diastolic diameter; LVEF, left ventricular ejection fraction; MAP, mean arterial pressure; NYHA: New York Heart Association; SD, standard deviation.

**Table 2:** Risk factors and types of devices in patients with LVAD using an ARNI.

Study	Risk Factors			Type of LVAD		
	Hypertension	Diabetes	Chronic Kidney Disease	HVAD	HM2	HM3
Alishetti et al., 2020 [13]	24 (80%)	12 (40%)	-	1 (3.3%)	12 (40%)	17 (57%)
Sharma et al., 2020 [16]	5 (100%)	3 (60%)	-	4 (80%)	1 (20%)	-
Zorz et al., 2020 [17]	-	-	-	-	-	-
Randhawa et al., 2020 [9]	-	-	-	-	-	-
Schnettler et al., 2021 [15]	-	-	-	-	-	-
Goldberg et al., 2021 [8]	-	-	-	-	-	-
Rawley et al., 2023 [14]	73 (88%)	-	42 (50.6%)	-	-	-

Data are presented as numbers (percentages). Abbreviations: HM2, Heartmate 2; HM3, Heartmate 3; HVAD, Heartware Ventricular Assist Device; LVAD, left ventricular assist device.

**Table 3:** Summary of included studies of patients with LVAD using an ARNI.

Study	Study Design	Follow-Up	Dose	Duration of ARNI	Types of Used LVAD	Device Strategy
Alishetti et al., 2020 [13]	Retrospective study	3 or 6 months	24–26 mg 49–51 mg 97–103 mg	Three and six months	HM2, HM3, HVAD	DT 16 (53.3%), BTT 13 (43.3%), BTR 1 (3.3%)
Sharma et al., 2020 [16]	Retrospective study	-	49 of 51 mg orally twice daily.	One month	HM2, HVAD	-
Zorz et al., 2020 [17]	Case series	6 months	-	six months	-	-
Randhawa et al., 2020 [9]	Case series	16 (7–20) days	low (≤24–26 mg, six patients) moderate (49–51 mg, four patients) The highest dose (97–103 mg) in four patients.	292 days (141–422)	Continuous flow-LVAD	DT (80%) Centrifugal (60%)
Goldberg et al., 2021 [8]	Case series	3 months	24/26 mg BID in 37%, up titrated to 49/51 mg BID in 44%, And reached 97/103 mg BID in 19%.	2018 to 2020	HM2 HM3 HVAD	-
Schnettler et al., 2021 [15]	Observational study	-	-	12 months	HVAD, HM3	-
Rawley et al., 2023 [14]	Cohort study	6 months	-	-	-	-

Data are presented as the number (percentage), mean ± SD, and median (IQR). Abbreviations: ARNI, angiotensin receptor–neprilysin inhibitor; BID, twice a day; BTR, bridge to recovery; BTT, bridge to transplant; DT, destination therapy; HM2, Heartmate 2; HM3, Heartmate 3; HVAD, Heartware Ventricular Assist Device; IQR, interquartile range; LVAD, left ventricular assist device; SD, standard deviation.

**Table 4:** Summary of the effect of an ARNI in patients with an LVAD.

Study	EF	CI	LVEDD	LVESD	BNP	MAP	NYHA Class	MPAP	Cr	CRP	GFR	Sodium	Potas-sium	BUN	BMI	Diuretic Dose
Alishetti et al., 2020 [13]	-	-	-	-	↓	↔	-	-	↔	↔	-	-	↔	↔	-	-
Sharma et al., 2020 [16]	-	-	-	-	-	↓	-	-	↔	-	↔	-	↔	-	-	-
Zorz et al., 2020 [17]	-	-	↔	-	↓	↔	-	-	↔	-	-	-	-	-	-	-
Randhawa et al., 2020 [9]	-	-	-	-	↔	↓	-	-	↔	-	-	-	↔	-	-	↔
Goldberg et al., 2021 [8]	-	-	↓	-	-	↓	↓	-	↔	-	-	↔	↔	↔	-	↔
Schnettler et al., 2021 [15]	-	-	-	-	-	-	-	-	-	-	-	-	-	-	-	-
Rawlley et al., 2023 [14]	↔	-	-	-	-	↔	-	-	-	-	-	-	-	-	↔	-

Abbreviations: BMI, body mass index; BNP, brain natriuretic peptide; BUN, blood urea nitrogen; CI, cardiac index; Cr, creatinine; CRP, C-reactive protein; EF, ejection fraction; GFR, glomerular filtration rate; LVEDD, left ventricular end-diastolic diameter; LVESD, left ventricular end-systolic diameter; MAP, mean arterial pressure; MPAP, mean pulmonary arterial pressure; NYHA class, New York Heart Association Class; ↓, decreased; ↔, unchanged.

## Data Availability

Not applicable.

## Supplementary Materials

The following supporting information can be downloaded at: https://www.mdpi.com/article/10.3390/jcm13247789/s1, Table S1: Search strategy of the studies in databases. Table S2: quality assessment of included studies using NOS and adapted NOS.

## Abbreviations

Abbreviation	Description
ACE-I	Angiotensin-Converting Enzyme Inhibitor
AKI	Acute Kidney Injury
ARB	Angiotensin II Receptor Blocker
ARNI	Angiotensin Receptor–Neprilysin Inhibitor
BID	Twice a Day
BMI	Body Mass Index
BNP	B-Type Natriuretic Peptide
BTR	Bridge to Recovery
BTT	Bridge to Transplant
BUN	Blood Urea Nitrogen
CI	Confidence Interval
Cr	Creatinine
DT	Destination Therapy
GFR	Glomerular Filtration Rate
HM2	HeartMate 2
HM3	HeartMate 3
HFrEF	Heart Failure with Reduced Ejection Fraction
HVAD	HeartWare Ventricular Assist Device
IQR	Interquartile Range
LVAD	Left Ventricular Assist Device
LVEDD	Left Ventricular End-Diastolic Diameter
LVEF	Left Ventricular Ejection Fraction
MAP	Mean Arterial Pressure
MPAP	Mean Pulmonary Arterial Pressure
NOS	Newcastle-Ottawa Scale
NYHA	New York Heart Association
PRISMA	Preferred Reporting Items for Systematic Reviews and Meta-Analyses
RAAS	Renin-Angiotensin-Aldosterone System
RCTs	Randomized Controlled Trials
SD	Standard Deviation
