# Supplementary material for: A Systematic Review and Meta-Analysis of an Angiotensin Receptor–Neprilysin Inhibitor in Patients Using a Durable Left Ventricular Assist Device
Source: J Clin Med. 2024 Dec 20;13(24):7789. doi: 10.3390/jcm13247789 (PMC11728431; doi:10.3390/jcm13247789)
Supplement: Supplementary file 1 [file jcm-13-07789-s001.zip › jcm-3301690-supplementary.pdf]

Supplementary file:

1. **Supplementary Table S1**: Search strategy of the studies in databases.
2. **Supplementary Table S2**: quality assessment of included studies using NOS and adapted NOS

Supplementary Table S1: Search strategy of the studies in databases.

PubMed	(((((((Entresto) OR (sacubitril/valsartan)) OR (LCZ696)) OR (Azmarda)) OR (Neparvis)) OR (angiotensin receptor/neprilysin inhibitor)) OR (ARNI)) AND ((((((((((((((((Left ventricular assist device) OR (lvad)) OR (heartmate)) OR (heartware)) OR (heart pump)) OR (jarvik-2000)) OR (thoratec)) OR (ventricular assist device)) OR (Vad)) OR (Mechanical Circulatory Support)) OR (mcs)) OR (biventricular assist device)) OR (bivad)) OR (Heart Assist Device)) OR (novacor)) OR (Left ventricular assist system)) OR (LVAS))	110
Scopus	(TITLE-ABS-KEY ((entrust OR "gabitril/valsartan" lcz696 OR amara OR hepavis OR angiotensin AND receptor/neprilysin AND inhibitor OR arnie)) AND ALL (("left ventricular assist device" OR lead OR heartwater OR heartwater OR "heart pump" OR jarvik-2000 OR thoriated OR "ventricular assist device" OR vad OR "Mechanical Circulatory Support" OR mcs OR "biventricular assist device" OR ivad OR heart AND assist AND device OR vacor OR "left ventricular assist system" OR lavas)))	91
Web Of Science	(entrepot OR “sacubitril/valsartan” OR LCZ696 OR armarda OR nepalis OR "angiotensin receptor/neprilysin inhibitor" OR ARNI) (All Fields) and ("left ventricular assist device" OR lvad OR heartmate OR heartware OR "heart pump" OR jarvik-2000 OR thoratic OR "ventricular assist device" OR vad OR "Mechanical Circulatory Support" OR mcs OR "biventricular assist device" OR bilad OR Heart Assist Device OR novaco OR "left ventricular assist system" OR lvad) (All Fields)	83

Cochrane Library	(Entresto OR “sacubitril/valsartan” OR LCZ696 OR Azmarda OR Neparvis OR “angiotensin receptor/neprilysin inhibitor” OR ARNI) AND ("left ventricular assist device" OR lvad OR heartmate OR heartware OR "heart pump" OR jarvik-2000 OR thoratec OR "ventricular assist device" OR vad OR "Mechanical Circulatory Support" OR mcs OR "biventricular assist device" OR bivad OR Heart Assist Device OR novacor OR "left ventricular assist system" OR LVAS)	18
Embase	('entresto'/exp OR entresto OR 'sacubitril/valsartan'/exp OR 'sacubitril/valsartan' OR 'lcz696'/exp OR lcz696 OR azmarda OR 'neparvis'/exp OR neparvis OR 'angiotensin receptor/neprilysin inhibitor' OR arni) AND ('left ventricular assist device'/exp OR 'left ventricular assist device' OR 'lvad'/exp OR lvad OR 'heartmate'/exp OR heartmate OR 'heartware'/exp OR heartware OR 'heart pump'/exp OR 'heart pump' OR 'jarvik 2000'/exp OR 'jarvik 2000' OR 'thoratec'/exp OR thoratec OR 'ventricular assist device'/exp OR 'ventricular assist device' OR vad OR 'mechanical circulatory support'/exp OR 'mechanical circulatory support' OR mcs OR 'biventricular assist device'/exp OR 'biventricular assist device' OR 'bivad'/exp OR bivad OR 'heart assist device'/exp OR 'heart assist device' OR (('heart'/exp OR heart) AND assist AND ('device'/exp OR device)) OR 'novacor'/exp OR novacor OR 'left ventricular assist system' OR lvas)	381
**Total**		683

Supplementary Table S2: quality assessment of included studies using NOS and adapted NOS

Study	Selection	Comparability	Outcome	Total score
Alishetti et al. 2022 (13)	3	-	2	5
Schnettler et al. 2021 (15)	3	2	3	8
Sharma et al. 2020 (16)	3	-	3	6
Zorz et al. 2020 (17)	3	-	2	5
Rawlley et al. 2023 (14)	4	1	3	8
Randhawa et al. 2020 (9)	2	-	3	5
Golderberg et al. 2021 (8)	3	-	3	6
